# Distinct endophytes are used by diverse plants for adaptation to karst regions

**DOI:** 10.1038/s41598-019-41802-0

**Published:** 2019-03-27

**Authors:** Fei Li, Xiaohong He, Yuanyuan Sun, Ximin Zhang, Xiaoxin Tang, Yuke Li, Yin Yi

**Affiliations:** 1 The Key Laboratory of biodiversity conservation in Karst mountain area of Southwest of china, Forestry Ministry, Guizhou Normal University, Guiyang, China; 2 Key Laboratory of Plant Physiology and Developmental Regulation, Guizhou Normal University, Guiyang, China; 3 School of Life Sciences, Guizhou Normal University, Guiyang, Guizhou China

## Abstract

The present study aimed at systematically investigating the endophytic communities of dominant plants in the karst ecosystem. Soil and plant materials were collected and after sequencing of the 16 s RNA, the diversity and abundance of the endophytic community structures in leaves were examined. Our results showed that abundant and diverse endogenous bacteria were associated with the leaves of common dominant plants living in the karst ecological environment. Notably, common traits and significant differences in the endophytic community structures were recorded among different plant species with different leaf grown in soils with different calcium contents. These observations implied that plants may adopt different strategies to adapt to the karst ecological environment. In addition, the endophytic bacteria associated with the leaves may be involved in different physiological strategies used by the plants to adapt to the karst ecological environment. These findings provide new avenues for developing microbial agents that could be suitable for the karst ecological environment and will provide sustainable solutions for improving the ability of plants to adapt to karst special adversities, and thus for karst geomorphological environmental protection and agricultural development.

## Introduction

Karst is a fragile and unique ecological environment and the primary rock of the karst topography is mainly composed of limestone, dolomite and other soluble carbonate rocks, covering the neutral to slightly alkaline soils rich in calcium and magnesium. The karst region is characterized by high calcium content (up to 1–3%), the average of which is several times higher than that of the non-karst regions. In addition, the ability of soils in karst regions for water conservation is extremely poor, which makes the soil surface very dry and cause serious water shortage in these regions. Another feature of the karst areas is that the nutritional potential of the soil is extremely poor due to the scarcity of nitrogen and phosphorus elements which are necessary for plant growth^1,2^.

The intake of calcium by plants is directly related to the exchangeable calcium content in the soil. The high calcium content of the soil causes the plant to absorb more calcium than normal, which leads to a number of serious consequences including hardening of the cell wall of the plant, inhibition of cell growth, disturbance of phosphoric acid-based energy metabolism, damage to the plant cell membrane structure and reduction of photosynthetic and transpiration rates, leading to leaf senescence^3,4^. Therefore, the plants that grow in the karst environment must have unique physiological and ecological adaptation mechanisms. Previous studies on the vegetation in the karst area showed that there are obligated calciferous plants that grow only in limestone areas^5^. Other researchers determined the calcium content and the exchangeable calcium content of the above ground and underground parts of common plants in various karst regions and found that 14 dominant species of karst ecosystem could be classified based on their aboveground and underground calcium content into high-calcium, low-calcium, and medium-calcium content plants^6,7^. High-calcium plants have a strong calcium-enrichment ability, and their above-ground parts can maintain high calcium content even in relatively low-calcium soils. The low-calcium plants have relatively low calcium content in their above-ground parts even in soils with high-calcium content. The medium-calcium content plants are case-dependent plants and their content in calcium is mainly affected by the exchangeable calcium content of the soil. These observations indicate that dominant plants living in the karst region adopt different ways to adapt to the high-calcium of the soil in these environments.

Endophytes plays important functions in the adaptation and evolution of plants to their environment. After 400 million years of evolution, there is a complete mechanism for the perception, signaling and response to external stress in the plant genome. However, most plants still cannot survive in highly hostile environments such as beaches and karst rocky desertification areas. Sufficient experimental evidence has proven that plants that grow in unfavorable environments such as salty, dry, hot and heavy metals-rich environments may develop different adaptation capacities to adversity and this may be partially due to symbiotic microorganisms^8–19^. All plants have been found to cohabit with endogenous microorganisms including bacteria, fungi, viruses, and microalgae^11–13^. These microorganisms are present in all plant organs including roots, stems, leaves, flowers, fruits and seeds, and are located in intercellular, xylem conduits and the intracellular parts of the plant cells^14,20^. Some scientists have even demonstrated that the phenotype of plants in nature is a product of the plant genome and their extensive interactions with microbial communities in the rhizosphere or *in vivo*^14^. Both greenhouse and field experiments have also confirmed that the removal of key endophytes can lead to the loss of resistance and adaptation abilities of some plants, which, for this reason, can no longer adapt to the unfavorable environment in which they originally used to live^8^. However, in the karst ecological environment, there has been no relevant study focused on the community structures and ecological functions of endophytic communities associated with dominant plants.

The dominant plants in the karst ecological environment include both gymnosperms and angiosperms which can physiologically adapt to high calcium and drought through a panoply of strategies. However, the similarities and differences in the endogenous microbial community structures of dominant plants in karst regions as well as the similarities and differences in the endogenous flora of the leaves of different plant species that adapt to the karst ecosystem with different mechanisms are unknown and needs an in-depth investigation.

In this study, we collected dominant plants and soil samples from several karst regions in Guizhou Province (China) to detect the endophytic bacterial community structures of the leaves of these dominant plants and identified bacterial communities common or specific to these plants that adapted to the karst ecosystem with different strategies. This work is the first systematic comparative study on the endophytic community structures associated with dominant plants in the karst ecological environment. Our findings will provide an avenue for the development of microbial agents that could be suitable for applications in the karst ecological environment and will provide sustainable solutions for improving the ability of plants to adapt to karst particular adversity and, thus, for the protection of karst topography and the development of agriculture in this kind of regions.

## Materials and Methods

### Collection of soils and plant materials

Samples were collected from karst regions in Guizhou Province from August to October 2017. According to different degrees of rocky desertification, Puding, Huajiang, Libo and Luodian regions located in the limestone mountain area of Xiaba Village, Maguan Town, Puding County, and the north plate of the Huaqiao Grand Canyon Iron Cable Bridge Scenic Area, 3 km south of Huajiang Town, Guanling County Jiangnan bank, Maobo karst forest nature reserve and LuodianQiandaohu wharf area were selected for the study. The degree of rocky desertification in the sampling sites in Puding and Huajiang was heavier, and the degree of rocky desertification in the sampling sites in Libo and Luodian was relatively light. The latitude and longitude of the sampling sites were as shown in Table 1. The collected samples were limited to small shrubs and herbaceous plants because small shrubs and herbaceous plants are the pioneer and dominant species in karst rocky desertification regions, and their use for research on the mechanism of plant adaptation to high calcium environment is representative and operational. Herbs and shrubs were collected in the local area as dominant or common species, and 3 plants were selected for normal growth. When collecting soil samples, the top soil, middle soil, and bottom soil distributed in the roots were collected and mixed in equal amounts.

**Table 1:** Data about the sample collection sites.

Sample number	Location	Latitude and longitude	Collection date	Altitude (m)	Soil calcium content (mg/kg)
1	Puding County	26°14.726′ N,105°45.507′ E	2017.08.06	1252	4289.89 ± 331.22
2	Guanling Yi and Miao Autonomous County	25°41.633′ N,105°36.746′ E	2017.08.08	950	3795.71 ± 250.91
3	Libo County	25°26.223′ N,108°7.117′ E	2017.08.10	550	3232.82 ± 146.04
4	Luodian County	25°25.441′ N,106°50.276′ E	2017.08.12	867	2547.53 ± 259.11

### Determination of total calcium content

For the determination of calcium content in plant leaves, 1.00 g of plant samples were digested with a concentrated H_2_SO_4_-H_2_O_2_ and filtrated. Then, 5 mL of filtrate was taken and added with 2 mL of 10% La(NO_3_)_3_ and diluted to 50 mL before determination of caclcium content by atomic absorption spectrophotometry.

For the determination of the exchangeable calcium content in soil samples, 10.00 g of air-dried soil were added with 50 mL of 1 mol·L^−1^ CH_3_COONH_4_ solution (pH 7.0) and shakeed at room temperature for 30 min. After filtering, 5 mL of filtrate was taken and added with 2 mL of 10% La(NO_3_)3. The content of exchangeable calcium was determined by atomic absorption spectrophotometry with a capacity of 50 mL.

### DNA extraction

To eliminate epiphytic microorganisms, the collected leaves of dominant plants were imbibed for 40 s in ethanol (75% vol/vol) and for 4 min in 1% sodium hypochlorite (vol/vol). Next, the leaves were washed thrice using sterile distilled water. The sterilization efficiency was confirmed by plating and culturing the sterile water used for rinsing (collected after the last washing) on LB medium. Subsequent experimental steps were followed only if no bacterial colony had grown on the LB medium, which was indicative of successful sterilization of the surface of the leaves. After sterilization, the leaves were soaked for 5 min in the DNA Away solution (DNA Away Surface Decontaminant; Thermo Fisher Scientific Inc, Waltham, MA, USA) to decontaminate leave surface from unwanted DNA and washed thrice using sterile water. After grinding of the leaves in presence of liquid nitrogen in frozen sterilized mortars with pestles, an aliquot of 100 mg leaf samples was taken for DNA extraction which was performed by using the CTAB method.

### 16S rDNA amplification and sequencing

For bacterial community analysis, the 16S rRNA gene sequencing was performed using the Illumina MiSeq sequencing platform. The primers used for the amplification of V4 region of the 16S rRNA gene were 799 F (5′-AAC AGG ATT AGA TAC CCT G-3′) and 1492 R (5′-GGT TAC CTT GTT ACG ACT T-3′) as forward and reverse primers, respectively. This primer pair features several mismatches with plant chloroplast 16S rRNA gene sequence, which prevent plant chloroplast from being sequenced (Wang *et al*. 2009). The first 10 cycles of PCR amplification were performed and the amplified products were then purified using AgencourtAmpure XP (Beckman Coulter, Inc., CA, USA). These amplification products were subsequently used as a template for the second PCR amplification using the same primers for 30 cycles. The thermocycler conditions were as follows: 94 °C 3 minutes, 94 °C 45 seconds, 50 °C 60 seconds, 72 °C 90 seconds, 72 °C 10 minutes and 4 °C holding. The PCR products were detected on a 1% agarose gel, and a negative control was used to confirm the absence of contamination. Positive amplicons were quantified using the PicoGreen dsDNA Assay Kit (Invitrogen, CA, USA), combined in equal amounts and then gel purified. The DNA library was sequenced using the Illumina MiSeq platform according to the manufacturer’s instructions^17^.

### Analysis of the sequencing data

The sequencing data was analyzed using the QIIME software package^18^ and UPARSE pipeline^19^. QIIME software was used for quality filtering of reads and processing with the default parameter settings for Illumina processing in QIIME (r = 3, p = 0.75 total read length; q = 3 and n = 0; herein, p is the minimum number of consecutive high-quality base reads to read, r the maximum allowed consecutive low-quality bases, in the maximum number of unrecognized (N) bases allowed in a sequence and q the considered as low-quality mass fraction). After assembling the sequences, UPARSE pipelines was used to obtain operational taxonomy units (OTUs). All sequences were assigned to OTUs with a similarity of 97%. We selected representative sequences for each OTU and used the Ribosomal Database Project (RDP) classifier^20^ to assign classification data to each representative sequence.

### Statistics and analysis

The following analyses were further performed on each sample 16S rRNA dataset obtained in the previous step: (i) Comparison of α-diversity (Chao value and Shannon index) and β-diversity (bray curtis algorithm) (ii) Significant test based on unpaired Student’s t-test and Wilcoxon Ranked test were performed to determine any differences between two variables and the significance of the differences was assessed using the FDR (false discovery rate). All of the above statistical analyses were performed using the R packages vegan and ggplot.

## Results

### Plants exhibit different mechanisms for adaptation to the karst environment

Four karst typical regions and six species of dominant plants growing in the karst ecological environment were selected and the calcium content in the leaves of these plants and the soils on which they live was evaluated. The data concerning the sampling sites and their locations are shown in Table 1. The measurement results were summarized in Table 2. The results showed that the calcium content of leaves from different dominant plants species and their corresponding rhizosphere soil displayed three different types of relationships that could be used for classifying the studied plants as low calcium, high calcium and environment-dependent plants. The low calcium plants were *Cyrtomiumfortunei* and *Pteris vittata*. The correlation coefficients between leaf calcium content and soil exchangeable calcium content were 0.1237 and −0.4067, respectively. Both of these plants, even in the high-calcium soil environment, maintained their leaf calcium content at a relatively low level (about 1% or less). High calcium plants comprised *Cayratia japonica* and *Ixerispolycephala*. The correlation coefficients of leaf calcium content and exchangeable calcium content in rhizosphere soil for these two plants were −0.5649 and −0.5327, respectively. Even in karst soils where the calcium content was relatively low, the calcium content of the leaves of these two plants remained at a high level (more than 2%). The dominant environment-dependent plants of the karst regions included the sweet potato *Ficustikoua* and *Bidenspilosavar*.*radiate*. In both plants, the leaf calcium content was closely correlated with the rhizosphere soil calcium content, and the correlation coefficients were 0.8388 and 0.9178, respectively.

**Table 2:** The relationship between soil and leaf calcium content.

Plant name	Growing Environment Soil Calcium Content (mg/kg)	Leaf calcium content (%)	Pearson Correlation coefficient	Adaptation type
*Cyrtomium fortunei*	4074.54 ± 524.40	0.734 ± 0.178	0.1237	Low calcium type (even though the soil calcium content is high, the leaves always maintain a low calcium content)
	3804.28 ± 331.77	1.053 ± 0.355		
	3150.17 ± 554.38	0.667 ± 0.284		
	2548.70 ± 600.35	0.857 ± 0.362		
*Pteris vittata*	4582.52 ± 470.33	0.387 ± 0.135	−0.4067	
	3724.80 ± 482.50	0.346 ± 0.082		
	3244.53 ± 380.23	0.656 ± 0.447		
	2743.76 ± 442.57	0.432 ± 0.155		
*Cayratia japonica*	4196.92 ± 447.29	2.882 ± 0.854	−0.5649	High calcium type (even though the soil calcium content is low, the leaves always maintain a high calcium content)
	4057.38 ± 265.74	3.484 ± 1.056		
	3319.54 ± 674.89	4.057 ± 1.135		
	2288.42 ± 573.48	3.653 ± 0.948		
*Ixeris polycephala*	3958.67 ± 624.40	1.838 ± 0.479	−0.5327	
	3544.80 ± 479.85	2.353 ± 0.769		
	3086.78 ± 772.84	2.664 ± 1.022		
	2548.64 ± 824.75	2.247 ± 0.862		
*Ficus tikoua*	4408.79 ± 580.46	2.342 ± 0.398	0.8388	Environment-dependent type (environmentally determined, leaf calcium content changes with soil calcium content).
	3816.49 ± 531.77	2.228 ± 0.482		
	3304.15 ± 910.27	2.265 ± 0.568		
	2586.75 ± 748.66	0.946 ± 0.435		
*Bidens pilosa* var.*radiate*	4517.88 ± 624.40	3.184 ± 0.637	0.9178	
	3826.53 ± 472.33	3.048 ± 0.652		
	3291.73 ± 875.49	2.062 ± 0.486		
	2568.92 ± 776.28	1.953 ± 0.506		

### The overall characteristics of endophyte community structure in leaves of dominant plants growing in karst regions

**Figure 1:**
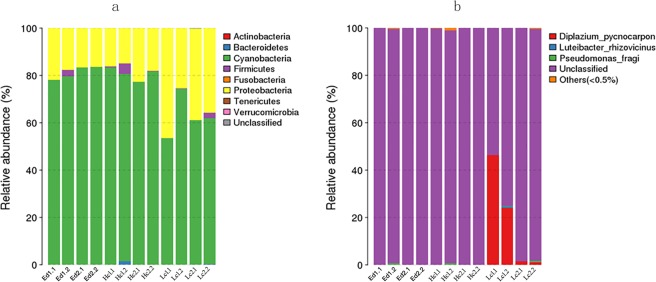
Endogenous bacterial community structure characteristics associated with each leaf sample. (**a**) Relative abundance of bacterial community at the phylum level. (**b**) Relative abundance of bacterial community at the species level. Ed: environment-dependent, Hc: high calcium, Lc: Low calcium, numbers infront of each samples indicate the sample number and replicate number.

Leaf endophytes play an important role in host adaptation to the environment. Thus, we aimed to investigate the similarities and differences in the community structure of endophytic bacteria in plants that adapt to different karst ecological environments. We selected two karst ecological environments with the highest and the lowest calcium levels (Puding County and Luodian County, Guizhou Province) and collected the above-mentioned six dominant plants species in these two karst regions. The characteristics of endogenous bacterial community structure in leaves were compared between these two regions, between different plant species and between different karst adaptation strategies. From all of the samples, we obtained a total of 1,025,220 high-quality sequences (read average length = 250 bp) with a minimum of 84,887 sequences per sample (Additional Table S1). Of the total number of sequences, 1,023,736 (99.85%) could be divided into 183 unique bacterial operational units (OTUs) at a sequence similarity level of 97%, averaging 46 OTUs per sample (Additional Table S2). All the OTUs obtained from all of the samples could be divided into 8 different bacterial phyla (Fig. 1a). The most abundant phylum was cyanobacteria, followed by proteobacteria. In all samples, the OTUs of these two bacterial phyla accounted for more than 90% of the bacterial community structure (Fig. 1a). The barplot of leaf bacterial species obtained from all of the samples was reported in Fig. 1b. The vast majority of the species were found as unclassified microorganisms (Fig. 1b). Among the classified species, *Diplazium pycnocarpon*, *Luteibacter rhizovicinus* and *Pseudomonas fragi* were the most representative species. The proportion of the other classified species was lesser than 0.5%. *D*. *Pycnocarpon* was more abundant in environment-dependent plant leaves compared to low calcium plant leaves (Fig. 1b).

### Different endophytic community structures are associated with plants that adapt to the karst environment

**Figure 2:**
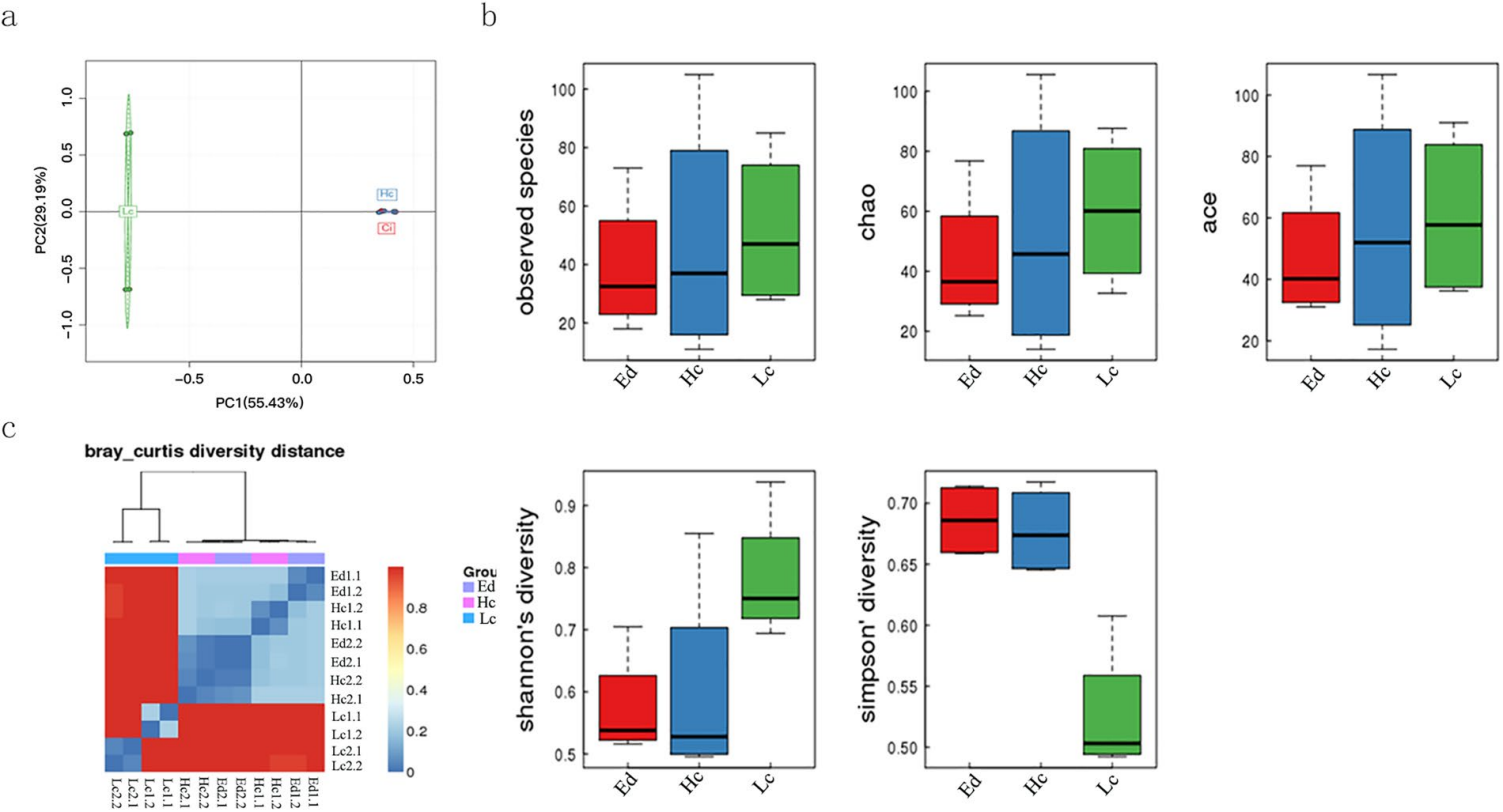
Different endophytic community structures are associated with plants that adapt to the karst environment. (**a**) Principal component analysis indicating the difference between samples based on the abundance of OTU. (**b**) Shannon and Simpson diversity analysis. (**c**) Bray Curtis similarity analysis of samples based on the OTU abundance. Ed: environment-dependent, Hc: high calcium, Lc: Low calcium, numbers infront of each samples indicate the sample number and replicate number.

We calculated the relative abundance of each OTU in each sample based on the abundance data of OTUs in each sample and used this abundance information to carry out principal component analysis (PCA) of the OTUs to identify the affected samples and the main factors for differences in bacterial community structure. We found that different adaptation strategies to the karst high-calcium environment were the main factors affecting the bacterial community structure (PC1) (Fig. 2a). In low calcium plants, the bacterial community structure was greatly affected by the calcium content of the soil (PC2). High calcium plants and environment-dependent plants had little differences in bacterial community structures. The species richness of the 12 samples was not significantly different, but the shannon index and simpson index (reflecting species diversity, including species richness and species evenness) showed that low calcium plants (*Cyrtomium fortunei* and *Pteris vittata*), high calcium plants (*Cayratia japonica* and *Ixeris polycephala*) and environment-dependent plants (*Ficus tikoua* and *Bidens ferulifolia*) had higher endogenous bacterial community diversity (Fig. 2b). Using the Bray–Curtis algorithm (Fig. 2c), the samples were subjected to cluster analysis and the distance between samples was calculated to determine the similarity of the species composition of each sample. Bacterial community showed a great difference between low calcium and environment-dependent plants while there was relatively little difference in endophytic bacterial community between high calcium and environment-dependent plants (Fig. 2c).

**Figure 3:**
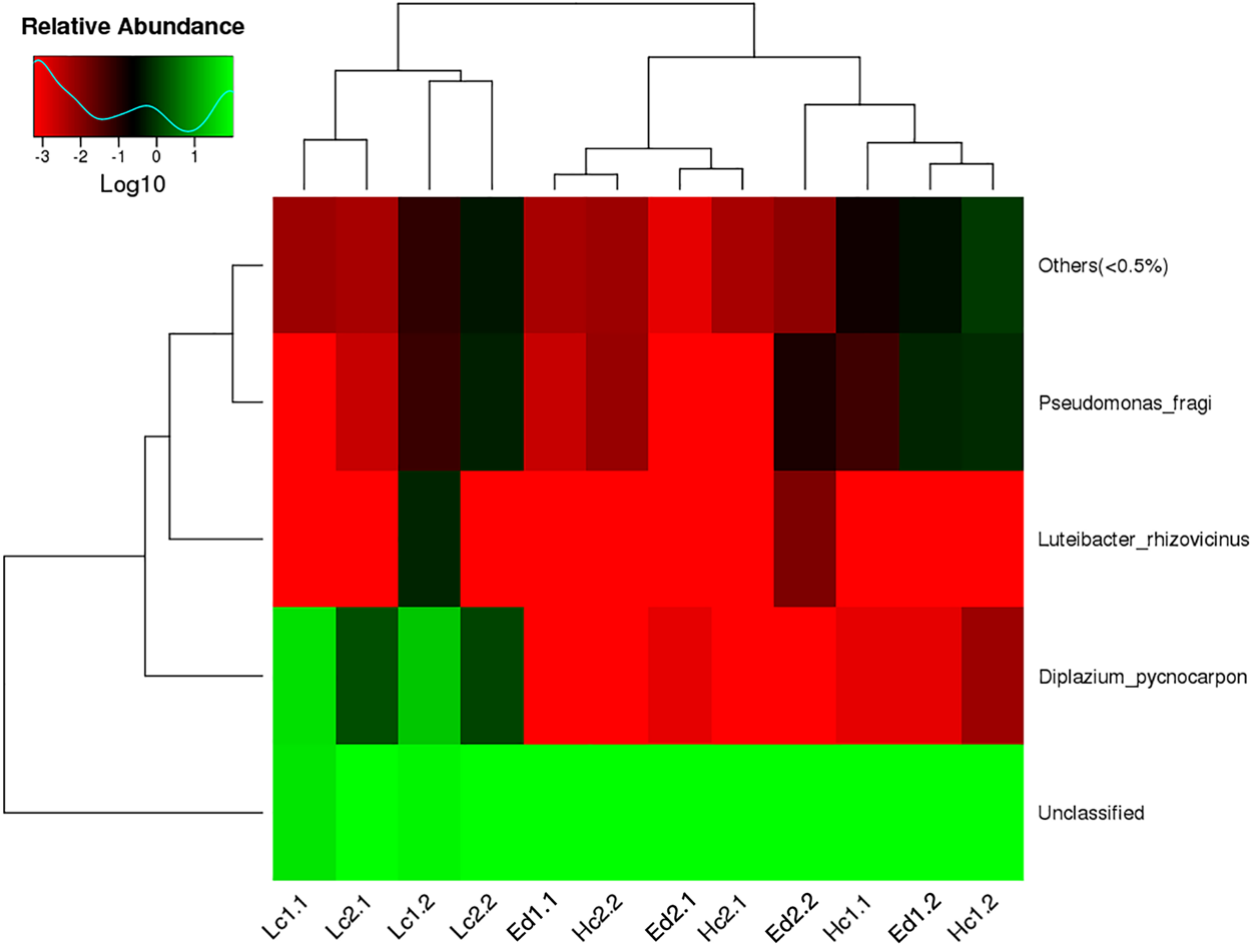
Heatmap showing the differences in bacteria between the endogenous bacterial communities of the leaves. Ed: environment-dependent, Hc: high calcium, Lc: Low calcium, numbers infront of each samples indicate the sample number and replicate number.

In order to identify the differences in bacteria between the endogenous bacterial communities of the leaves, we used statistical methods to examine the differences in the abundance of microbial communities between the samples and assessed the significance of the differences using the FDR (false discovery rate). From the test results, species that led to differences in sample composition could be screened. The genus Diplazium belonging to the Alphaproteobacteria was the most abundant in the low calcium plants (*Cyrtomium fortunei* and *Pteris vittata*) and the high calcium plants (*Cayratia japonica* and *Ixeris polycephala*) but its presence was extremely low in the leaves of environment-dependent plants (*Ficus tikoua* and *Bidens Pilosa var*.*radiate*) (Fig. 3).

### Differences in Plant Bacterial community Owing to Different Strategies Used for Adapting to Karst Eco-environment

**Figure 4:**
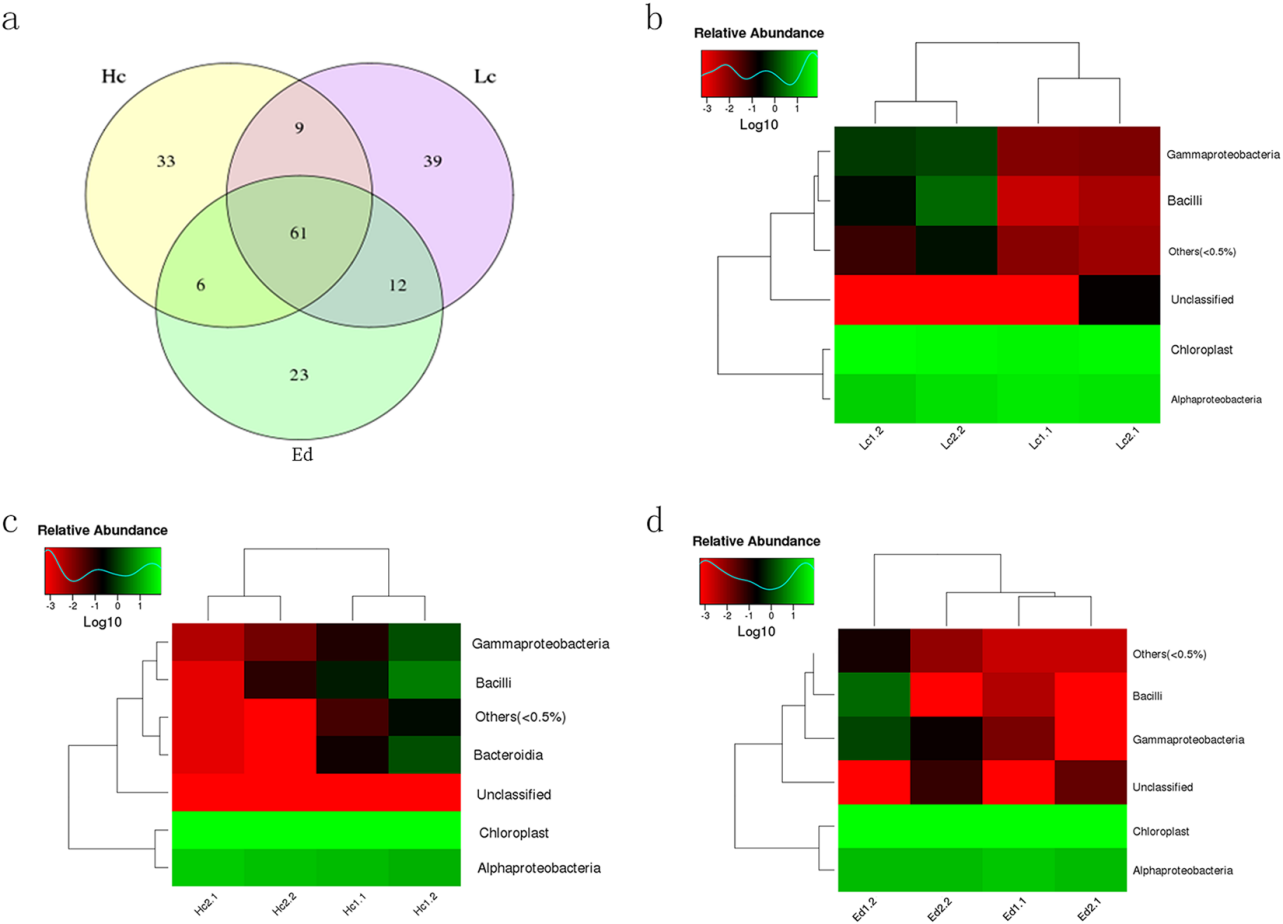
Diversity of endophytes associated with the leaves of the different dominant plants in the karst regions. (**a**) Venn diagram showing the abundance of OTUs associated with each type of leaves and the number of OTUs shared among them. (**b**) Heatmap showing the OTUs abundant in low calcium plant leaves. (**c**) Heatmap showing the OTUs abundant in high calcium plant leaves. (**d**) Heatmap showing the OTUs abundant in environment-dependent plant leaves. Ed: environment-dependent, Hc: high calcium, Lc: Low calcium, numbers infront of each samples indicate the sample number and replicate number.

We use GLMM (general linear mixed model) to analyze all samples and identify the differential OTUs between samples. There were 61 OTUs common to karst dominant plant leaves (Fig. 4). Thirty-nine OTUs specifically appeared in the endophytic community of leaves of low calcium plants (Additional Table S3). The abundance of OTUs in endophytic bacterial communities of low calcium plants was higher than that in high calcium and environment-dependent plants (Fig. 4a, Additional Table S3). In addition, 6 OTUs appeared in the leaves of high calcium and environment-dependent plants and were absent from the leaves of the low calcium plants (Fig. 4a, Additional Table S3). We further analyzed the classification of these differential OTUs (Fig. 4b–d). The OTUs specific to the leaves of low calcium plant were different from those specific to the leaves of high calcium and environment-dependent plants. In soils with different calcium levels, the differential OTUs included specific bacteria. The above observations indicated that the effect of calcium content on the bacterial community structures was relatively weak.

## Discussion

Because of its high calcium content, drought and poor content in nutritional elements, the karst ecological environment is extremely fragile. Once the ground vegetation is destroyed, it will cause water and soil loss, resulting in surface rocky desertification and the regional environment will no longer be suitable for human survival, and the environmental recovery will become extremely difficult. This feature of the karst ecological environment can also cause countless problems to the local economic development. In order to preserve the soil as much as possible and avoid soil erosion, local farmers must spend enormous manpower and material resources to transform the slopes into terraced fields. Comparatively to plain areas, agricultural production in terraced fields is arduous due to the difficulty to use large machineries, the extremely high cost of cultivation and the lower yield.

There are some common dominant plants in the karst ecological environment. Some scholars have classified plants on karst topography according to their dependence on limestone soils. They are roughly divided into: 1) Calciphiles which are distributed only on limestone soils; 2) Calcioles which can grow normally on high-calcium soil while a few of them can grow on acid soil; 3) Calcifuges, which are only distributed on acidic soils and cannot grow normally on limestone soils; 4) Calcium-indifferent plants, which are distributed in limestone and acid soils, are insensitive to soil calcium content^5^. This classification reveals the distribution of plants in the karst ecological environment but does not reflect the adaptation mechanism of plants to high-calcium karst environments. In corroboration with previous studies, our findings indicated significant differences in the calcium content and distribution of different types of plants, suggesting different patterns of calcium absorption and utilization by different plant species. The calcium content of leaves of some plants was significantly correlated with the soil exchangeable calcium content, but calcium content of leaves of other plants had no correlation with the soil exchangeable calcium content (Table 2), indicating that the adaptation of karst plants to high calcium environment were not similar. Thus, plants may adopt a diversity of mechanisms to adapt to high calcium environments. Plant leaves are the main site of plant physiological activities; thus, the calcium content of plant leaves reflects the amount of calcium required for plant physiological activities. In this study, based on the correlation between the calcium content of the plant and its correlation with the soil calcium content, the analysis of strategies used for plant adaptation to high calcium environment was conducted. The dominant plants in the karst region could be classified into low calcium, high calcium and environment-dependent plants (Table 2). The different correlations between leaf calcium content and soil calcium content suggested that these three types of plants adapted to karst regions using different strategies. The leaf and soil calcium contents also affected the endogenous bacterial community structures of the leaves. Since microbial communities play important roles in plant physiological and metabolic processes, we anticipated that these endophytes may be involved in the adaptation strategies adopted by plant growing in the karst ecosystems. This provided a basis for further exploration of the mechanisms used by plants to adapt to high calcium content in karst regions.

Endophytes are important determinants of host plant resistance to stress^8^. Endogenous bacteria have a short reproductive cycle and a rapid evolution, which can help the host plant adapt to new environments in a quick manner^21^. Compared to transformation of functional gene to improve plant tolerance, inoculation with beneficial microorganism presents significant advantages. First of all, growth-promoting microorganisms can be conveniently transferred to diverse kinds of plants to improve the stress resistance of inoculated plants^22,23^. Secondly, beneficial microorganisms often confer to the host the ability to cope with multiple adversities^24,25^. Compared with transgenic pathways that can only improve the traits of plants, beneficial microbial infection pathways endow significant production and ecological applications, especially for the complex high calcium karst environment. The present work is the first systematic study on the endophyte community structures associated with plants in the karst ecological environment. The results showed that there were abundant and diverse endogenous bacteria in the leaves of common dominant plants living in the karst ecological environment. There was some notable similarities and differences in the endophytic bacterial community structures among different plant species with different calcium content and among different plant species with different adaption strategies to the karst ecological environment.

The important role of endogenous bacteria in host plant adaptation to local environment has received growing attention and the endogenous bacterial communities of model plants and important crops have been already studied. These studies have shown that important factors influencing plant endophytic bacterial community structures include soil physicochemical properties, host plant species and plant organs^26,27^. In the present study, the endophytic community structures of leaves were studied based on the physiological characteristics of plants, microbial species or soil properties. We found that in the dominant plants living in karst regions, distinct microbial community structures were associated with plant leaves. Previous studies demonstrated that bacterial community structures play important roles in plant growth, resistance to diseases and different environmental stresses^28–31^. Based on these studies, we suggest that, in karst regions, soil calcium content influences the bacterial communities and that different bacterial communities may participate in physiological strategies used by the plants to adapt to the karst ecological environment. These findings provide a new perspective for examining the ecological roles of endophytic bacterial community structures and the influencing factors.

The differential OTUs and the corresponding bacteria may exert some effects on host plants. Different physiological strategies used by plants to adapt to the karst ecological environment, the endogenous bacteria that exist in the leaves, the specific mechanism of the physiological processes of the plant will be the focus of our next research. This study will open an avenue for the development of microbial agents that are suitable for the ecological environment of karst regions and will provide sustainable solutions for improving the plant ability to adapt to karst particular adversity, and thus for the protection of karst topography and the development of agriculture.

## Supplementary information

SUPPLEMENTARY DATA

## Data Availability

All data generated or analysed during this study are included in this published article.
