# Supplementary material for: Distinct endophytes are used by diverse plants for adaptation to karst regions
Source: Sci Rep. 2019 Mar 27;9:5246. doi: 10.1038/s41598-019-41802-0 (PMC6437200; doi:10.1038/s41598-019-41802-0)
Supplement: Supplementary file 1 — SUPPLEMENTARY DATA [file 41598_2019_41802_MOESM1_ESM.docx]

**SUPPLEMENTARY DATA**

**Distinct endophytes are used by diverse plants for adaptation to karst regions**

Fei Li^#,1^, Xiaohong He^# ,1^, Yuanyuan Sun ^1^, Ximin Zhang ^2^, Xiaoxin Tang^2^, Yuke Li^1^, Yin Yi *^,3^

^1^The Key Laboratory of biodiversity conservation in Karst mountain area of Southwest of china, Forestry Ministry, Guizhou Normal University

^2^Key Laboratory of Plant Physiology and Developmental Regulation, Guizhou Normal University

^3^School of Life Sciences, Guizhou Normal University, Guiyang, Guizhou, China

^#^: Both authors contributed equally to this work.

***Correspondence:** Yin Yi, School of Life Sciences, Guizhou Normal University, Guiyang550001, China. Phone/: +(86)851-86766891, Fax: +(86)851-86766891, Email: [yiyin1964@outlook.com](mailto:yiyin1964@outlook.com).

**TABLE OF CONTENTS**

**Additional Table S1. Summary of Sample's Reads and Tags.** A total of 1025220 pairs of clean reads, corresponding to an average read number of 85435±417, and a total of 1023736 connect tags corresponding to an average of 85311±440 connect tags with an average tag length of 252 ± were obtained from the 12 analyzed samples.

**Additional Table S2. The OTUs statistics.** A total of 183 OTUs corresponding to 1011335 sequences were obtained from the 12 samples. The minimum number of OTUs per sample was 11 while the maximum number was 105 with an average of 46 OTUs per sample.

**Additional Table S3. Lists of OTUs obtained from each sample and comparison between groups.**

**Additional Table S1. Summary of Sample's Reads and Tags.** A total of 1025220 pairs of clean reads, corresponding to an average read number of 85435±417, and a total of 1023736 connect tags corresponding to an average of 85311±440 connect tags with an average tag length of 252 ± were obtained from the 12 analyzed samples.

| #Sample Name | Total Pairs Read Number | Connect Tag Number | Connect Ratio (%) | Average Length And SD | Tags Without Primer | Tag Utilization Ratio (%) | Average Length And SD |
| --- | --- | --- | --- | --- | --- | --- | --- |
| Ed1.1 | 85751 | 85616 | 99.84 | 252/1 | - | - | -/- |
| Ed1.2 | 85121 | 85010 | 99.87 | 252/1 | - | - | -/- |
| Ed2.1 | 84962 | 84714 | 99.71 | 252/1 | - | - | -/- |
| Ed2.2 | 85452 | 85077 | 99.56 | 252/1 | - | - | -/- |
| Hc1.1 | 85113 | 85023 | 99.89 | 252/1 | - | - | -/- |
| Hc1.2 | 85572 | 85477 | 99.89 | 252/1 | - | - | -/- |
| Hc2.1 | 86240 | 86141 | 99.89 | 252/1 | - | - | -/- |
| Hc2.2 | 85123 | 85055 | 99.92 | 252/1 | - | - | -/- |
| Lc1.1 | 85382 | 85342 | 99.95 | 253/1 | - | - | -/- |
| Lc1.2 | 84887 | 84796 | 99.89 | 253/0 | - | - | -/- |
| Lc2.1 | 85956 | 85884 | 99.92 | 253/2 | - | - | -/- |
| Lc2.2 | 85661 | 85601 | 99.93 | 253/1 | - | - | -/- |

**Additional Table S2. The OTUs statistics.** A total of 183 OTUs corresponding to 1011335 sequences were obtained from the 12 samples. The minimum number of OTUs per sample was 11 while the maximum number was 105 with an average of 46 OTUs per sample.

| Sample name | Tag number | OTU number |
| --- | --- | --- |
| Ed1.1 | 84503 | 18 |
| Ed1.2 | 83791 | 73 |
| Ed2.1 | 84020 | 28 |
| Ed2.2 | 83718 | 37 |
| Hc1.1 | 83005 | 53 |
| Hc1.2 | 84138 | 105 |
| Hc2.1 | 84966 | 11 |
| Hc2.2 | 84375 | 21 |
| Lc1.1 | 85111 | 31 |
| Lc1.2 | 84028 | 63 |
| Lc2.1 | 85146 | 28 |
| Lc2.2 | 84534 | 85 |
| Total |  | 553 |
| Mean |  | 46 |

**Additional Table S3. Lists of OTUs obtained from each sample and comparison between groups.**

| group | Share OTU_num/unique OUT num | OUT ID |
| --- | --- | --- |
| Hc-vs-Lc | 70 | Otu1,Otu10,Otu100,Otu102,Otu104,Otu106,Otu108,Otu109,Otu111,Otu113,Otu120,Otu128,Otu13,Otu131,Otu132,Otu14,Otu140,Otu143,Otu146,Otu149,Otu15,Otu153,Otu155,Otu16,Otu160,Otu165,Otu166,Otu17,Otu170,Otu176,Otu18,Otu181,Otu182,Otu19,Otu2,Otu22,Otu23,Otu26,Otu3,Otu32,Otu33,Otu34,Otu35,Otu36,Otu37,Otu4,Otu40,Otu42,Otu43,Otu48,Otu5,Otu50,Otu55,Otu59,Otu6,Otu65,Otu7,Otu71,Otu75,Otu78,Otu8,Otu83,Otu85,Otu86,Otu87,Otu91,Otu93,Otu94,Otu96,Otu98 |
| Hc-vs- Ed | 67 | Otu1,Otu10,Otu100,Otu102,Otu104,Otu106,Otu108,Otu111,Otu113,Otu117,Otu120,Otu123,Otu13,Otu131,Otu14,Otu140,Otu146,Otu149,Otu15,Otu153,Otu155,Otu16,Otu160,Otu162,Otu165,Otu166,Otu168,Otu17,Otu170,Otu176,Otu179,Otu18,Otu181,Otu182,Otu19,Otu2,Otu22,Otu23,Otu26,Otu3,Otu32,Otu33,Otu35,Otu36,Otu37,Otu42,Otu43,Otu48,Otu5,Otu50,Otu55,Otu59,Otu6,Otu65,Otu7,Otu71,Otu78,Otu8,Otu83,Otu86,Otu87,Otu91,Otu93,Otu94,Otu96,Otu98,Otu99 |
| Lc-vs-Ed | 73 | Otu1,Otu10,Otu100,Otu102,Otu104,Otu106,Otu108,Otu111,Otu113,Otu119,Otu12,Otu120,Otu13,Otu131,Otu137,Otu14,Otu140,Otu141,Otu146,Otu149,Otu15,Otu150,Otu153,Otu155,Otu16,Otu160,Otu164,Otu165,Otu166,Otu17,Otu170,Otu176,Otu18,Otu181,Otu182,Otu19,Otu2,Otu22,Otu23,Otu26,Otu29,Otu3,Otu31,Otu32,Otu33,Otu35,Otu36,Otu37,Otu42,Otu43,Otu44,Otu48,Otu5,Otu50,Otu51,Otu55,Otu59,Otu6,Otu65,Otu69,Otu7,Otu71,Otu78,Otu8,Otu83,Otu86,Otu87,Otu9,Otu91,Otu93,Otu94,Otu96,Otu98 |
| Hc-vs-Lc-vs- Ed | 61 | Otu1,Otu10,Otu100,Otu102,Otu104,Otu106,Otu108,Otu111,Otu113,Otu120,Otu13,Otu131,Otu14,Otu140,Otu146,Otu149,Otu15,Otu153,Otu155,Otu16,Otu160,Otu165,Otu166,Otu17,Otu170,Otu176,Otu18,Otu181,Otu182,Otu19,Otu2,Otu22,Otu23,Otu26,Otu3,Otu32,Otu33,Otu35,Otu36,Otu37,Otu42,Otu43,Otu48,Otu5,Otu50,Otu55,Otu59,Otu6,Otu65,Otu7,Otu71,Otu78,Otu8,Otu83,Otu86,Otu87,Otu91,Otu93,Otu94,Otu96,Otu98 |
| Hc | 33 | Otu101,Otu103,Otu105,Otu11,Otu110,Otu114,Otu115,Otu116,Otu118,Otu121,Otu127,Otu129,Otu133,Otu134,Otu135,Otu138,Otu147,Otu151,Otu152,Otu159,Otu163,Otu172,Otu173,Otu178,Otu183,Otu184,Otu185,Otu39,Otu52,Otu64,Otu66,Otu70,Otu72 |
| Lc | 39 | Otu112,Otu125,Otu126,Otu130,Otu136,Otu142,Otu144,Otu148,Otu158,Otu161,Otu171,Otu174,Otu175,Otu177,Otu180,Otu20,Otu24,Otu28,Otu38,Otu41,Otu45,Otu47,Otu49,Otu53,Otu57,Otu58,Otu60,Otu63,Otu73,Otu76,Otu77,Otu79,Otu81,Otu82,Otu84,Otu88,Otu90,Otu92,Otu95 |
| Ed | 23 | Otu107,Otu124,Otu139,Otu145,Otu154,Otu156,Otu157,Otu167,Otu169,Otu21,Otu25,Otu27,Otu30,Otu54,Otu56,Otu61,Otu62,Otu67,Otu68,Otu74,Otu80,Otu89,Otu97 |
